# Managing Mortality and Aging Risks with a Time-Varying Lee–Carter Model

**DOI:** 10.3390/healthcare11050743

**Published:** 2023-03-03

**Authors:** Zhongwen Chen, Yanlin Shi, Ao Shu

**Affiliations:** 1 College of Economics and Management, Hengyang Normal University, Hengyang 421002, China; 2 Department of Actuarial Studies and Business Analytics, Macquarie University, Sydney, NSW 2109, Australia; 3 Business School, Hunan University, Changsha 410012, China

**Keywords:** mortality rates, Lee–Carter model, time-varying coefficients, rotated age pattern, life expectancy

## Abstract

Influential existing research has suggested that rather than being static, mortality declines decelerate at young ages and accelerate at old ages. Without accounting for this feature, the forecast mortality rates of the popular Lee–Carter (LC) model are less reliable in the long run. To provide more accurate mortality forecasting, we introduce a time-varying coefficients extension of the LC model by adopting the effective kernel methods. With two frequently used kernel functions, Epanechnikov (LC-E) and Gaussian (LC-G), we demonstrate that the proposed extension is easy to implement, incorporates the rotating patterns of mortality decline and is straightforwardly extensible to multi-population cases. Using a large sample of 15 countries over 1950–2019, we show that LC-E and LC-G, as well as their multi-population counterparts, can consistently improve the forecasting accuracy of the competing LC and Li–Lee models in both single- and multi-population scenarios.

## 1. Introduction

The ongoing improvement of human life expectancy around the world has far-reaching influences on many aspects of our society. For instance, Maestas et al. [1] highlight the importance of considering demographic changes, including mortality projections, in analyzing labor supply. Mortality projections are used to predict future changes in the size and age structure of the labor force, which can help inform policy decisions related to workforce development and training, immigration, and other factors that affect the labor market. For the healthcare system, Fenton et al. [2] indicate the importance of considering changes in mortality projections in analyzing healthcare utilization and expenditure. Specifically, prediction of future demand for health and medical care services is dependent on mortality projections. Such predictions may inform policy decisions related to the allocation of resources for healthcare and the design of related systems. The importance of accurate mortality projections is also witnessed in retirement and pensions and the insurance industry (see, for example, ref. [3], among others).

To better model the underlying patterns of mortality improvement is, therefore, essential to enhance accuracy of mortality projection. Among the existing studies, Lee and Carter [4] is the mostly widely used factor-based mortality model, known as the LC model. Despite its popularity, the LC model assumes constant age-specific mortality decline speeds over time. Therefore, the LC model produces increasingly large proportional differences in the projected death rates, even at adjacent ages, in the long run. Specifically, the projected mortality rates of LC are implausibly low for infants and younger ages relative to older ages. This is in contrast to the findings in influential studies, including Li et al. [5], that found that mortality declines at younger ages have been slowing while declines at older ages have been accelerating.

A “rotation” approach of age-specific mortality decline speed was later proposed by Li et al. [5] to enhance the LC model. However, this rotation is of an ad hoc nature rather than being data-driven [6]. To address this, we investigate a time-varying coefficients extension of the LC model. Simply speaking, we employ a local constant kernel smoothing method [7] to model and forecast dynamic age-specific mortality declines. In this paper, we consider two commonly adopted kernel functions: Epanechnikov and Gaussian, and the corresponding extensions of the LC model are denoted by LC-E and LC-G, respectively. Technical details of the modelling approach are provided, and our empirical results consider data of 15 low-mortality populations sourced from the famous Human Mortality Database [8], including annual mortality rates of age 0–100 spanning 1950–2019.

The contributions of this paper are threefold. First, the proposed time-varying coefficients extension of the LC model effectively complements the rotation adopted in Li et al. [5]. The proposed flexible framework ensures that the speed of rotation is data-driven, rather than ad hoc. The existence of closed-form solutions further makes the proposed extension computationally efficient. Secondly, using a comprehensive population dataset, we systematically demonstrate the outstanding performance of the time-varying models. The overall superiority of the LC-E and LC-G models is robust when the age and temporal setting are changed. The analysis of life expectancies up to 2100 further shows that the LC-G model can well address the issue of underestimated mortality improvements at old ages, existing for the LC model [5]. Thus, the LC-E and LC-G models may provide potentially more accurate forecasts than the LC model in the research regarding long-term mortality projection. Finally, we describe the multi-population extensions of the employed models and illustrate their forecasting performances. Consistent with the single-population case, the newly proposed multi-population extensions are still overall the best performing model among all investigated specifications.

The rest of this paper is structured as below. Section 2 describes the data and models. We present empirical results with discussions in Section 3. Section 5 concludes the paper.

## 2. Data and Methods

### 2.1. Database

To study the mortality rates, the data of this paper are sourced from the famous Human Mortality Database [8], or HMD. HMD provides detailed mortality and population data for over 40 developed countries. We employ the annual age-specific central mortality rates, which are derived from official statistics from each country. HMD is maintained by the Max Planck Institute for Demographic Research in Rostock, Germany, and the University of California, Berkeley, United States of America (USA). As stated in its official website, the purpose of HMD is to make mortality data available for scientific research and to promote research in social sciences, such as actuarial studies, demographics, labor economics, and public health and care.

In order to study the consistent mortality declines, we explore 15 low-mortality countries as considered in Li and Lee [9]: Australia (instead of West Germany), Austria, Canada, Denmark, the United Kingdom, Finland, France, Italy, Japan, the Netherlands, Norway, Spain, Sweden, Switzerland, and the USA. For the low-mortality nature, mortality rates are more likely to consistently decline across all ages. This enables one to observe the potential rotating patterns on age-specific mortality rates, as argued by Li et al. [5]. Following Booth et al. [10], we choose an opportune range of data starting from 1950 to 2019 in order to have a reliable and complete dataset. The crude uni-sex (total) mortality data for ages 0–100 are studied in this paper.

**Figure 1:**
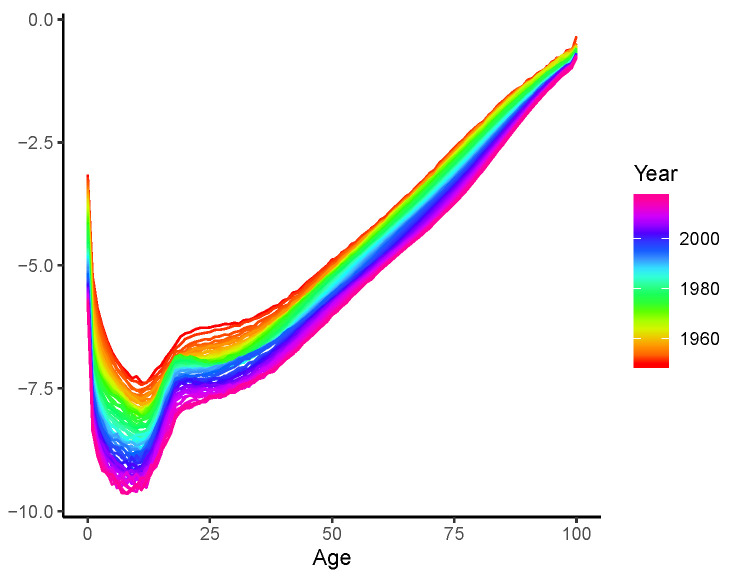
Averaged logged mortality rates.

We present logged mortality rates averaged across the 15 populations in Figure 1. Consistent mortality declines are observed at all ages over time. Appropriate modelling of those patterns is critical to credible mortality projections, which is the key to the accurate actuarial, demographic, and healthcare practices (see, for example, refs. [11,12,13,14], among others).

### 2.2. The Lee–Carter and Related Models

Suppose we have mortality data of *N* ages, each age with *T* years of observations. The Lee–Carter (LC) model (1992) ([4]) summarizes the systematic mortality trends of the *N* ages by a common factor. Formally, the log central mortality rate at age *x* in year *t*, $\ln m_{x,t}$, follows the specification given by:

(1) $$\ln m_{x,t}=a_{x}+b_{x}k_{t}+\varepsilon_{x,t},$$

where $a_{x}$ is the mortality level, i.e., the average mortality rate over time at age *x*, $k_{t}$ is the period effect, i.e., the systematic mortality trend common to all ages, $b_{x}$ is the age effect at *x*, i.e., the sensitivity of $\ln m_{x,t}$ to $k_{t}$, and $\varepsilon_{x,t}$ is the normal residual term with mean 0 and variance $\sigma_{\varepsilon_{x}}^{2}$. As noted by [4], Equation (1) is not identifiable without normalization constraints. For example, one could multiply $b_{x}$ with a constant *c* and divide $k_{t}$ by the same constant and reach the same fitting results. In [4], the following normalization constraints are imposed:

$$\sum_{t}k_{t}=0,\mathrm{and}\sum_{x}b_{x}=1.$$

Given the constraint of $k_{t}$, $a_{x}$ is set to the mean of $\ln m_{x,t}$ over the sample considered. The LC model is then estimated by singular value decomposition (SVD) instead of the usual ordinary least square approach in the original paper. (A maximum likelihood estimation method may also be employed to calibrate the parameters [15]. Alternatively, a Bayesian approach is proposed in Li et al. [16]. Compared to those estimation methods, SVD is much more computationally efficient and stable, which is critical to the proposed time-varying framework of this paper.)

While $a_{x}$ and $b_{x}$ are assumed to be constant, the period effect is often modeled by a time-series process. In particular, ref. [4] assumes a random walk with drift specification, which is adopted by many later studies:

(2) $$k_{t}=k_{t-1}+d+e_{t},$$

where the drift term *d* measures the average annual change in $k_{t}$, and $e_{t}\overset{i.i.d.}{∼}N\left(0,\sigma_{e}^{2}\right)$. Based on the time-series specification in Equation (2), future mortality rates can be projected by extrapolating the period effect $k_{t}$. Specifically, the expected *h*-step-ahead mean forecasts of the period effect and the log central death rate are given by:

(3) $$\begin{array}{cc} {\hat{k}}_{T+h} & =k_{T}+hd,\\ \ln{\hat{m}}_{x,T+h} & ={\hat{a}}_{x}+{\hat{b}}_{x}{\hat{k}}_{T+h}, \end{array}$$

where *T* is the last year of the sample.

**Remark** **1**. *Since the forecast ${\hat{k}}_{T+h}$ is just a linear trend, Booth et al. [17] consider two adjustments in estimating the optimal ${\hat{k}}_{t}$ to fit this trend. First, the modeling of $k_{t}$ is modified by considering the age distribution of deaths via a Poisson model. Second, a method is implemented to identify the most appropriate fitting period to address potential departure of the linearity of $k_{t}$. The resulting extension is known as the Booth–Maindonald–Smith (BMS) model. In addition, the influential work of Brouhns et al. [18] points out the inappropriateness of using the simple SVD with Gaussian errors when dealing with mortality data. The first adjustment of the BMS model essentially estimates the drift term $d_{k}$ in Equation (2) in a generalized linear model with Poisson family, which effectively resolves this issue. Consequently, we adjust $d_{k}$ as in the BMS approach for all models considered in this paper.*

### 2.3. Issues with the Lee–Carter Model and Related Literature

**Figure 2:**
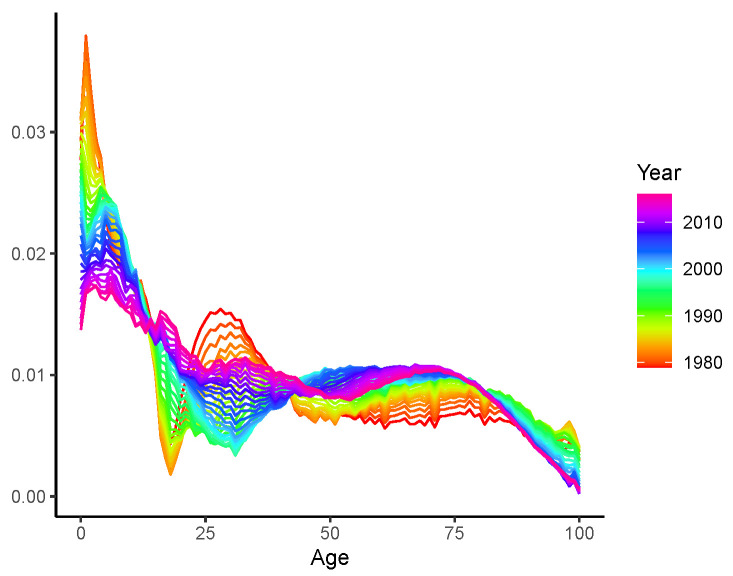
Dynamic age-specific declines ${\hat{b}}_{x,t}$.

For the long-spanning nature of mortality data, the static parametric structure of the LC model may not be compatible with reliable long-term demographic research. To see this, the specification of the LC model decomposes mortality improvements into the product of latent non-stationary mortality trend factors ($k_{t}$), assumed as a random walk with drift, and the corresponding age specific loadings of these trends ($b_{x}$), assumed constant over time. Considering the data displayed in Figure 1, we plot the fitted $b_{x}$ using a rolling window of a 30-year width in Figure 2. (There are no available data for the first 29 time points (i.e., 1950–1978) using the rolling window approach.) Clearly, the fitted $b_{x}$ becomes roughly “flatter” over time, with decreasing (increasing) weights for the younger (older) ages. Ignoring such a dynamic pattern therefore reduces the credibility of the forecast mortality rates and life expectancies of LC. Even when the BMS model is used, the static parametric nature is not necessarily sufficient to address this issue.

The insufficiency of the static $b_{x}$ over time is recognized in the influential work by Li et al. [5]. To resolve it, a “rotation” approach is proposed, which specifies that the out-of-sample ${\hat{b}}_{x}$ will be dynamic and gradually converge to an ultimate structure. However, such an ultimate structure is determined by expert (ad hoc) judgments rather than being data-driven [6].

### 2.4. A Time-Varying Coefficients Extension of the Lee–Carter Model

To systematically address the ad hoc issue of Li et al. [5], we investigate a time-varying coefficients extension of the LC model. Specifically, we employ a local constant kernel smoothing method [7], also known as the Nadaraya–Watson estimator, to accommodate the dynamics in the age-specific mortality declines. A novel application of such an estimator in mortality forecasting can be found in Chang and Shi [19]. This approach allows $b_{x}$ to be time-dependent over both the in-sample (denoted by $b_{x,t}$) and out-of-sample (denoted by $b_{x,T+h}$) periods. In this paper, we consider two commonly adopted kernel functions: Epanechnikov and Gaussian, and the corresponding extensions of the LC model are denoted by LC-E and LC-G, respectively. The technical details are described in this section.

#### 2.4.1. A Time-Varying Framework Using Kernels

To incorporate the time-varying coefficients, we extend the LC model as follows:

(4) $$\ln m_{x,t}=a_{x}+b_{x,t}k_{t}+\varepsilon_{x,t},$$

such that the age-specific declines are allowed to be dynamic. This suggests that the relative age-specific mortality decline speed is now time-dependent. In terms of the forecasting, we have that

(5) $$\ln{\hat{m}}_{x,T+h}={\hat{a}}_{x}+{\hat{b}}_{x,T+h}{\hat{k}}_{T+h},$$

where ${\hat{b}}_{x,T+h}$ is the forecast $b_{x,t}$ at step *h*, and ${\hat{k}}_{T+h}$ is identically defined as in Equation (3). Consequently, to obtain forecasts of this time-varying framework, one needs to additionally model and forecast the time-dependent $b_{x,t}$. (Note that we do not require the assumption $b_{x,t}>0$. Negative values mean that mortality rates at certain ages may (temporarily) increase (relative to other ages), rather than decline. As explained above, since this paper works with low-mortality populations, consistent declines are expected and more likely to occur.

For the estimation of the time-varying $b_{x,t}$, many effective methods have been discussed in the statistics literature, including polynomial splines [20,21], smoothing splines [22,23], and kernel-local polynomial smoothing [24]. Among them, one of the most widely used methods is the kernel-local polynomial smoothing, which includes the local constant and the local linear estimation. In this paper, we illustrate our method using the local constant kernel smoothing method [7], also known as the Nadaraya–Watson estimator.

**Remark** **2**. *Regarding the selection of kernels, an influential criterion is to compare the resulting asymptotic mean integrated squared error. Among all popular kernels, Epanechnikov minimizes this error and is therefore recognized as the most efficient kernel [25]. The Epanechnikov kernel is also investigated in various mortality research including Chang and Shi [19]. For comparison purposes, the Gaussian kernel is also investigated, which is widely used in modern machine learning methodology. The Gaussian kernel is especially popular for techniques involving the principal component analysis (related to the SVD in LC), for its attractive technical features. See Theodoridis [26] for a review on this topic.*

To implement the estimation, at each time *t*, the usual SVD will be applied once to $K_{b}\left(\tau_{s}-\tau \right)\ln m_{x,s}$, where s=1,2,...,T, τ=t/T and $\tau_{s}=s/T$. The resulting estimated “weighted” age-specific declines will be the corresponding ${\hat{b}}_{x,t}$. In addition, $K_{b}\left(\tau_{s}-\tau \right)$ is a known kernel function with *b* known as the bandwidth, and $K_{b}\left(\tau_{s}-\tau \right)$ is essentially a weight for the *s*th observation. For instance, with the widely employed Gaussian kernel, we have that

$$K_{b}\left(\tau_{s}-\tau \right)=\frac{1}{\sqrt{2\pi }b}e^{-\frac{{\left[\left(\tau_{s}-\tau \right)/b\right]}^{2}}{2}}$$

which is essentially the Gaussian density evaluated at $(\tau_{s}-\tau )/b$ and further scaled by *b*. The bandwidth *b* then determines the weights distributed to each observation when estimating $b_{x,t}$. A larger *b* means weights are more evenly distributed. Other than the Gaussian kernel, we also examine the popular Epanechnikov kernel in this paper, which is specified as

$$K_{b}\left(\tau_{s}-\tau \right)=\frac{0.75}{b}{\left\{1-{\left[\left(\tau_{s}-\tau \right)/b\right]}^{2}\right\}}_{+}.$$

This kernel is also investigated in various mortality research, including Chang and Shi [19]. In this paper, we denote the time-varying coefficients LC model estimated by the Gaussian and Epanechnikov kernels as LC-G and LC-E, respectively.

**Figure 3:**
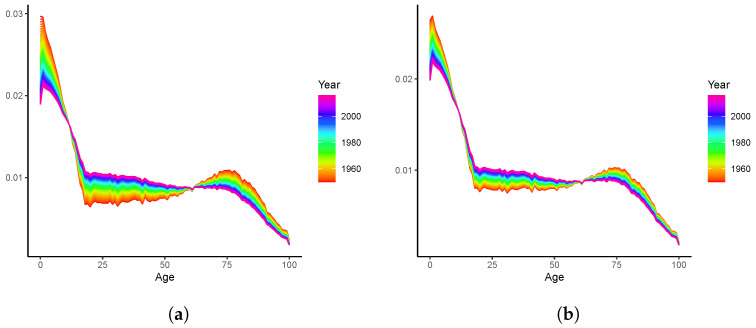
Fitted age-specific declines ${\hat{b}}_{x,t}$ of the averaged population, 1950–2019: (**a**) Epanechnikov kernel; (**b**) Gaussian kernel.

We now employ the LC-E and LC-G models to revisit the mortality rates presented in Figure 1, and the resulting fitted ${\hat{b}}_{x,t}$ are plotted in Figure 3a,b, respectively. respectively. (The bandwidths are chosen in a similar manner as that described in Section 2.4.3. In short, we employ the same hold-out sample strategy for data spanning 1950–2019. Forecasts of the age-specific mortality declines (${\hat{b}}_{x,t}$) are produced using the exponential smoothing state space model discussed in Feng and Shi [27]. The bandwidths are then chosen as those minimize the forecasting errors of logged mortality rates in the hold-out sample). Both the fitted curves and time-varying patterns of the LC-E and LC-G models are similar to each other. Compared to the preliminary results displayed in Figure 1b with a running window, less randomness is observed. This may be explained by the fact that the entire (weighted) sample is used to produce each individual ${\hat{b}}_{x,t}$. Roughly speaking, dynamics produced by kernels and the running window are consistent. However, despite that we do observe the rotation of ${\hat{b}}_{x,t}$ over most age groups as argued by Li et al. [5], the mortality declines at very old ages seem to decelerate rather than accelerate. Thus, an adjustment needs to be adopted before reliable mortality modelling and forecasting can be implemented with the dynamic ${\hat{b}}_{x,t}$. Otherwise, the forecast mortality improvements at old ages may gradually decline, leading to a larger gap between mortality rates of relatively younger and older ages. This is contrary to the rotation proposed by Li et al. [5] and the concept of (age) coherence studied in the demographic and actuarial literature (see, for example, [6,9,28], among others).

#### 2.4.2. Coherent Modelling of the Dynamic Age-Specific Mortality Declines

To adjust ${\hat{b}}_{x,t}$, we require that forecast mortality rates across ages will not diverge in the long run. This idea, known as age coherence, is much in-line with the rotation argued in Li et al. [5] and is critical to producing biologically reasonable forecasts of mortality rates in the long-term analysis [6]. The age coherence is formally defined as follows.

**Definition** **1**. *Age coherence means that for the h-step-ahead forecasts, $|\ln{\hat{m}}_{i,T+h}-\ln{\hat{m}}_{j,T+h}|=O_{p}\left(1\right)$, ∀i,j∈(1,...,N), where if $X_{n}=O_{p}\left(a_{n}\right)$ then for any ε>0, there exists a finite M>0 and a finite N>0 such that $P(|X_{n}/a_{n}|\geq M)\leq \varepsilon$∀n>N. That is, when h→∞, $|\ln{\hat{m}}_{i,T+h}-\ln{\hat{m}}_{j,T+h}|$ will not diverge to infinity.*

To accommodate the age coherence in mortality forecasting, it is worth noting that $\ln m_{x,t}$ is usually assumed to be a non-stationary I(1) process (see, for example, [6,19,28,29,30], among others). Thus, let $y_{x,t}=\ln m_{x,t}-\ln m_{x,t-1}$, which is known as the mortality improvement of age *x* at time *t*, its long-run mean must be time-independent due to the stationarity. It can then be shown that

$$\ln{\hat{m}}_{x,T+h}=E\left(\ln m_{x,T+h}|\Omega_{T}\right)=\ln m_{x,T}+\sum_{k=1}^{h}E\left(y_{x,T+k}|\Omega_{T}\right)=O_{p}\left(1\right)+h{\hat{\mu }}_{x}$$

when *h* goes large, where $\Omega_{T}$ is the information set at time *T*. Thus, we have that

$$|\ln{\hat{m}}_{i,T+h}-\ln{\hat{m}}_{j,T+h}|=O_{p}\left(1\right)+\left|h\left({\hat{\mu }}_{i}-{\hat{\mu }}_{j}\right)\right|,$$

where ${\hat{\mu }}_{x}$ is the fitted long-run mean of the stationary series $y_{x,t}$. It can then be seen that the only scenario to ensure the age coherence is that ${\hat{\mu }}_{i}\equiv {\hat{\mu }}_{j}$ for all i,j=1,...,N. From Equation (5), when h→∞, it is easy to see that ${\hat{y}}_{x,T+h}={\hat{b}}_{x,T+h}d$, where *d* is identically defined as in Equation (2). Further, since $y_{x,t}$ is assumed stationary, the condition of ${\hat{\mu }}_{i}\equiv {\hat{\mu }}_{j}$ is equivalent to ${\hat{b}}_{i,T+h}\equiv {\hat{b}}_{j,T+h}$ for all i,j=1,...,N. With the constraint of the LC model, such that $\sum_{x}{\hat{b}}_{x,T+h}=1$, we require that ${\hat{b}}_{x,T+h}$ converges to 1/N for all x=1,...,N when h→∞.

We now discuss an appropriate model to study $b_{x,t}$ such that the age coherence of the forecast rates is ensured. Note that the condition all ${\hat{b}}_{x,T+h}$ converging to the same constant 1/N implies that $b_{x,t}-1/N$ is a stationary sequence with 0 mean for all *x*. Hence, we denote this sequence by $b_{x,t}^{*}$, an appropriate parametric structure analogous to that discussed in Li and Lu [6] and described below.

(6) $$\begin{array}{cc} b_{1,t}^{*} & =\alpha_{1}b_{1,t-1}^{*}+\varepsilon_{1,t}^{b}\\ b_{2,t}^{*} & =\alpha_{2}b_{2,t-1}^{*}+\beta_{2}b_{1,t-1}^{*}+\varepsilon_{2,t}^{b}\\ b_{i,t}^{*} & =\alpha_{i}b_{i,t-1}^{*}+\beta_{i}b_{i-1,t-1}^{*}+\gamma_{i}b_{i-2,t-1}^{*}+\varepsilon_{i,t}^{b} \end{array}$$

where i=3,...,N, and t=1,...,T. Essentially, Equation (6) is a vector autoregressive (VAR) model with constrained coefficient matrix. According to Li and Lu [6], the constraint is to emphasize the impact of the temporal effect (measured by $\alpha_{i}$) and those of the cohorts effect (measured by $\beta_{i}$ for the same cohort and $\gamma_{i}$ for the nearest younger cohort). The forecasts of ${\hat{b}}_{x,T+h}^{*}$ are produced in the same way as for a usual VAR model, based on which we can derive ${\hat{b}}_{x,T+h}={\hat{b}}_{x,T+h}^{*}+1/N$. It can be seen that as long as the VAR of Equation (6) is stationary (a sufficient condition would be that summations of absolute values of $\alpha_{i}$, $\beta_{i}$, and $\gamma_{i}$ are all smaller than or equal to 1 for all i=1,...,N), ${\hat{b}}_{x,T+h}^{*}$ will converge to 0 for all *x* in the long run, and thus the forecast mortality rates will be age coherent. In addition, at each forecasting step, we can rescale ${\hat{b}}_{x,T+h}$ to sum up to 1 to satisfy the constraint $\sum_{x}{\hat{b}}_{x,T+h}=1$.

In terms of the estimation, Equation (6) can be solved using the penalized least squares (PLS), and its loss function is defined below:

(7) $$\begin{array}{cc} LF= & \sum_{i=3}^{N}\sum_{t=2}^{T}{\left[b_{i,t}^{*}-\alpha_{i}b_{i,t-1}^{*}-\beta_{i}b_{i-1,t-1}^{*}-\gamma_{i}b_{i-2,t-1}^{*}\right]}^{2}+\\ & \sum_{t=2}^{T}\left[b_{2,t}^{*}-\alpha_{2}b_{2,t-1}^{*}-\beta_{2}b_{1,t-1}^{*}\right]+\sum_{t=2}^{T}{\left(b_{1,t}^{*}-\alpha_{1}b_{1,t-1}^{*}\right)}^{2}+\\ & \lambda_{\alpha }\sum_{i=2}^{N}{\left(\alpha_{i}-\alpha_{i-1}\right)}^{2}+\lambda_{\beta }\sum_{i=3}^{N}{\left(\beta_{i}-\beta_{i-1}\right)}^{2}+\lambda_{\gamma }\sum_{i=4}^{N}{\left(\gamma_{i}-\gamma_{i-1}\right)}^{2}, \end{array}$$

where $\lambda_{\alpha }$, $\lambda_{\beta }$, and $\lambda_{\gamma }$ are pre-selected smoothing parameters of $\alpha_{i}$, $\beta_{i}$, and $\gamma_{i}$, respectively.

**Remark** **3**. With greater values of those λ’s, the fitted coefficients will be smoother across ages. When all λ’s are equal to 0, this is a special case of PLS with no penalty and reduces to the usual ordinary leas square case. With more smoothly changed coefficients, the forecast ${\hat{b}}_{i,T+h}^{*}$ is expected to vary smoother from i−1 to i. This aims to reduce the roughness among fitted and forecast $b_{i,t}^{*}$, which is inevitable since mortality data are often of a small sample size [6]. The quadratic nature of LF, ${\hat{\alpha }}_{i}$, and ${\hat{\beta }}_{i}$ will have closed-form solutions, which are computationally efficient. Nevertheless, it is important to note that both LC-E and LC-G models will introduce additional 3N−3 parameters in the estimation, compared to the original LC model.

#### 2.4.3. Tuning Parameters Selection and the Estimation Procedure

The only outstanding issue now is how to select the tuning parameters, i.e., smoothing penalties of the time-varying coefficients LC models. In a usual case, a cross-validation technique can be employed to prevent the model from overfitting. It is worth noting that a usual cross-validation technique for time-series data, such as the expanding-window approach explained in Hyndman and Athanasopoulos [31], is not quite applicable in our case. The reason is that this expanding-window approach normally considers a very short forecasting step. In contrast, age-coherent adjustment of the LC model emphasizes the long-term forecast. Thus, we employ a hold-out-sample approach to select the tuning parameters (*b* and three λ’s), which minimizes

$$\mathrm{RMSFE}=\sqrt{\frac{1}{N(T/3)}\sum_{i=1}^{N}\sum_{h=1}^{T/3}{\left(\ln{\hat{m}}_{i,2T/3+h}-\ln m_{i,2T/3+h}\right)}^{2}},$$

where RMSFE is the root of mean squared forecasting errors (since logged mortality rates are modeled, the RMSFE also considers the logged rather than original values. If an exponential transformation is applied, the predicted original mortality rates are unavoidably exposed to the famous Jensen’s inequality issue. Thus, the accuracy of forecasting is inherently lower than the logged rate is considered), and the evaluation period is given by the last third ([2T/3,T]) of the data in our study. (Note that the choice of the length of the test sample (one-third) is common among existing studies. Adopting other popular alternatives, such as the last fourth, fifth, and tenth sample will lead to robust results.)

In summary, forecasting with the LC-E and LC-G models can be implemented with the following steps:

1. Fit the original LC model with the full sample to obtain ${\hat{a}}_{x}$, ${\hat{k}}_{T}$, and *d*;
2. For the training sample spanning [1,2T/3−1], use the Epanechnikov (LC-E) or Gaussian (LC-G) kernel to obtain $b_{x,t}$, based on which the constrained VAR model of Equation (6) is fitted for $b_{x,t}^{*}$;
3. Select the optimal tuning parameters *b*, $\lambda_{\alpha }$, $\lambda_{\beta }$, and $\lambda_{\gamma }$ via a grid search, such that the RMSFE of the test sample over [2T/3,T] is minimized;
4. With the selected tuning parameters, fit the LC-E or LC-G model for the full sample;
5. Forecast ${\hat{b}}_{x,T+h}$ with the fitted model, and then produce $\ln{\hat{m}}_{x,T+h}$ with Equation (5), where ${\hat{a}}_{x}$ and ${\hat{k}}_{T+h}={\hat{k}}_{T}+hd$ sourced from Step 1.

To study the uncertainties of the forecast, the same procedure of the LC model can be applied for the LC-E and LC-G models proposed in this section. For instance, assuming that $e_{t}$ in Equation (2) follows a Gaussian distribution, the prediction interval (PI) of the estimated life expectancies will be produced based on simulations. More specifically, we use method (I) described in Li [32] to implement the simulation, and a 95% PI is then comprises the 2.5th and 97.5th percentiles of the simulated results.

**Remark** **4**. *The proposed specification and estimation of the LC-E and LC-G models have four advantages. First, from an empirical point of view, this is in line with the rotation investigated in Li et al. [5] for finite steps of forecasting. More specifically, the convergence of ${\hat{b}}_{x,T+h}$ to an identical constant enables mortality decline to decelerate at younger ages and accelerate at older ages, which effectively adjusts the inconsistence observed in Figure 1b and Figure 3. Second, unlike that determined by expert opinions as in Li et al. [5], the speed of rotation/convergence is data-driven for both LC-E and LC-G and is therefore not ad hoc. Third, the computational cost is low for the existence of closed-form solutions compared to other potentially more flexible but (much) more computationally intensive parametric frameworks. Fourth, different from the ultimate pattern of ${\hat{b}}_{x,T+h}$ investigated in Li et al. [5], the rotations achieved by the LC-E and LC-G models realize the age coherence in the long run. This ensures the non-divergence of mortality rates across all ages, which is biologically reasonable and thus theoretically appealing.*

### 2.5. Related Mortality Models

Other than the three factors ($a_{x}$, $b_{x}$, and $k_{t}$) used in the LC model, existing research has proposed alternative specifications to model mortality rates. For instance, Renshaw and Haberman [15], Cairns et al. [33], Renshaw and Haberman [34], and Plat [35] have considered additional factors, such as the cohort impacts and/or other temporal factors. Brouhns et al. [18], Currie et al. [36], Wang and Lu [37], and Debon et al. [38] calibrate mortality rates with different statistical methodologies. Machine learning techniques are considered in Hainaut [39], Levantesi and Pizzorusso [40], Nigri et al. [41], and Doukhan et al. [42]. Other studies, such as [43,44,45], introduce different time-series models to forecast the temporal patterns. Comprehensively reviewing those models is out of the scope of this paper, and please see specifications therein for details.

## 3. Results and Discussion

**Figure 4:**
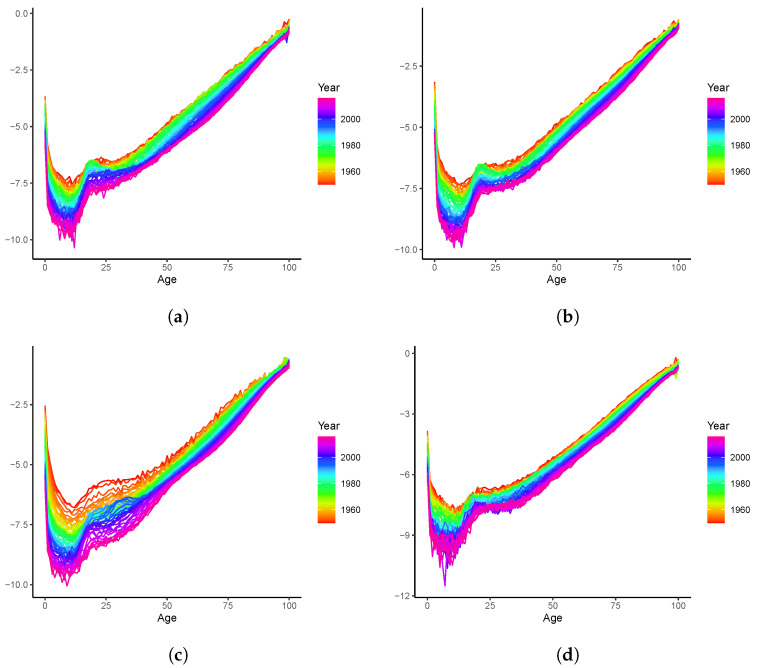
Mortality rates ($\ln m_{x,t}$) over 1950–2019:(**a**) Australia; (**b**) Canada; (**c**) Spain; (**d**) Sweden.

We model the mortality data and present the results in this section. As demonstrated in Figure 1, on average, the mortality rates consistently decline across all ages. This is also true for individual populations, as can be observed in Figure 4, where four countries are inspected for illustrative purposes. Despite the data volatility in some cases (e.g., Sweden, due to its small population size), substantial mortality improvements take place for both young and old ages. For instance, the “accidental hump” of ages 20–25 which was outstanding in the 1980s, has been flattening over time in all countries.

Following the steps described in Section 2, we fit all models over the dataset 1950–2000 as the training set, and forecast mortality rates from 2000 to 2019 as the test set. The selected bandwidths are displayed in Table 1 for the LC-E and LC-G models. To ensure the validity of the VAR(1) model to study $b_{x,t}$, the assumptions of stationarity and adequacy of VAR(1) need to be tested for the in-sample ${\hat{b}}_{x,t}$. The I(0) feature is tested via the usual augmented Dickey–Fuller (ADF) unit root test, and the adequacy of VAR(1) is tested by the Granger causality (GC) test. Recall that for each population, we have a total of 101 age groups, and the $b_{x,t}$ is estimated in 2 sets via the LC-E and LC-G. All tests are performed at the 5% level, and the number out of 101 violated assumptions are presented in Table 1. It is clear that in the vast majority of cases, both the stationarity and model adequacy assumptions hold. The actual percentages of negative outcomes are close to expected (i.e., 5%). Those results validate the application of the VAR(1) framework for both the LC-E and LC-G models.

### 3.1. Out-of-Sample Forecasting Analysis

We firstly consider the forecasting performance of the proposed LC-E and LL-G models. In particular, we compare their out-of-sample forecasting accuracy with those of the LC and BMS models. Following Li and Lu [6], Feng et al. [28], and Chang and Shi [19], recall that the training sample is set to 1950–2000, and the test sample is 2001–2019. To measure the accuracy, the RMSFE is computed as follows, where the mortality data of each country are fitted into each of the four models individually:

$${\mathrm{RMSFE}}_{h}=\sqrt{\frac{1}{101\times h}\sum_{i=1}^{101}\sum_{s=1}^{h}{\left(\ln{\hat{m}}_{i,2000+s}-\ln m_{i,2000+s}\right)}^{2}}.$$

Thus, RMSFE${}_{19}$ is the overall forecasting accuracy measure covering the entire test period, whereas RMSFE${}_{h}$ with 1≤h≤19 is the accumulated forecasting accuracy measure up to the *h*th step.

Our baseline results, including single-year age mortality rates over 1950–2019, are presented in Table 2. Overall, except for the UK data, the RMSFE${}_{19}$ of LC-E and LC-G are uniformly smaller than those of the LC model for all the examined populations. This suggests the improved forecasting performance considering the time-varying ${\hat{b}}_{x,T+h}$. The BMS model, with adjustments on both shortened training sample period and estimation of $k_{t}$, improves the forecasting accuracy of LC in seven countries. Despite this, except for the UK, Japan, the Netherlands and the USA, both the LC-E and LC-G models consistently outperforms the BMS counterparty in 13 cases. This suggests that appropriately allowing for dynamic age-specific mortality declines is more effective than the two adjustments in BMS to improve the forecasting accuracy. Although the LC-E model outperforms LC-G in six cases, the differences between the two approaches are marginal. This indicates that the forecasting performance of the time-varying coefficients framework described in Section 2.4 is robust against specific kernel functions. Finally, the RMSFE${}_{19}$ averaged over the fifteen countries of the LC, BMS, LC-E, and LC-G models are 0.2576, 0.2610, 0.2202, and 0.2197, respectively. Thus, on average, BMS cannot improve the forecasting performance of LC, whereas the LC-E and LC-G models can improve it by 14.7%. From a statistical point of view, if an unknown population is randomly chosen, we expected that LC-E or LC-G is more likely to improve the out-of-sample forecasting, with the forecasting error expected to be reduced by roughly 15%.

**Figure 5:**
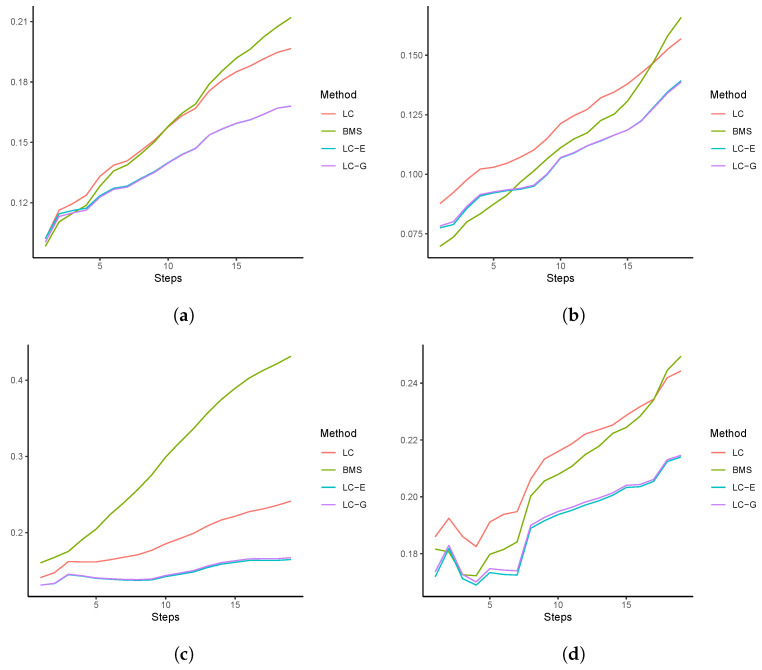
RMSFE over forecasting steps: (**a**) Australia; (**b**) Canada; (**c**) Spain; (**d**) Sweden.

To gain more insights of the four models’ performances at different horizons, we plot the RMSFE${}_{h}$ at each of the sixteen forecasting steps. For demonstration purposes, those of Australia, Canada, Spain, and Sweden are presented in Figure 5. Results of other countries are available upon request. In the vast majority of cases, we see that the RMSFEs of the LC-E and LC-G models are smaller than those of the other two models. This indicates that LC-G and LC-H are able to generate relatively more accurate forecasting results at different horizons.

We now evaluate the robustness of the forecasting results by performing the out-of-sample forecasting analysis under two major variant settings: (1) we follow Li et al. [5] and model the logged mortality rates of the five-year ages instead of the single-year groups; and (2) we consider a shorter training sample over 1970–2000 to avoid fluctuations in 1950–1969 for certain populations (e.g., Spain). A summary of the RMSFE${}_{19}$ across the 15 countries is reported in Table 3. Overall, we conclude that both LC-E and LC-G are able to produce satisfying forecasting performances that are robust across different settings. In addition, using different kernel functions in the time-varying framework only leads to marginal differences in the out-of-sample forecasts.

### 3.2. Long-Term Analyses: 2020–2100

**Figure 6:**
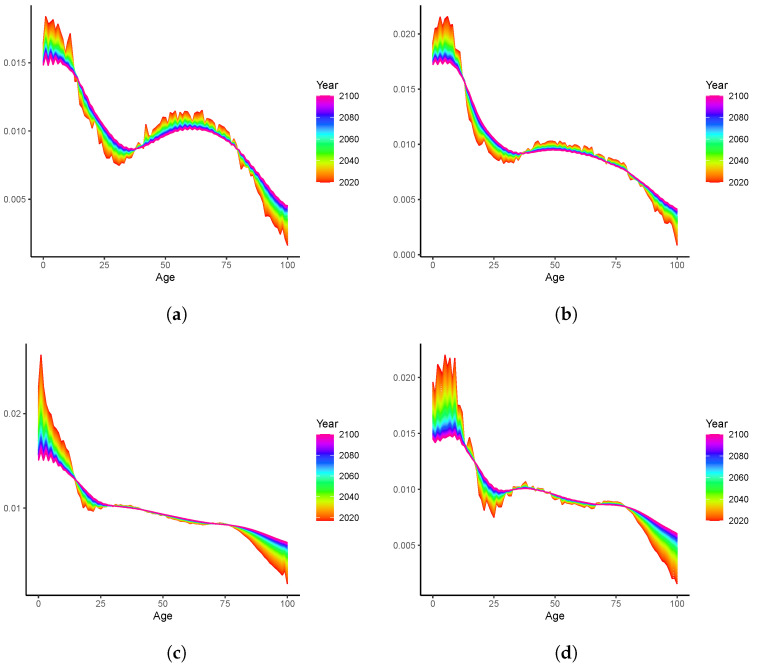
Projected rotation of age-specific mortality decline (${\hat{b}}_{x,T+h}$), 2020–2100: (**a**) Australia; (**b**) Canada; (**c**) Spain; (**d**) Sweden.

We now conduct long-term analyses beyond the full sample period using the proposed time-varying LC models. Since the produced forecasts are robust against the selected kernels, it is sufficient to focus on one model only. More specifically, we follow the steps described in Section 2.4.3 to fit the LC-G model using the full sample (1950–2019) and produce ${\hat{b}}_{x,T+h}$ over 2020–2100. We report the forecasts for Australia, Canada, Spain, and Sweden in Figure 6. Overall, all ${\hat{b}}_{x,T+h}$ are converging to the long-run mean 1/101. In other words, all the curves are “rotating” to a flat horizontal line. The dynamics are consistent with those of Li et al. [5] (see, for example, Figure 5), with younger ages faster and older ages slower. Unlike in Li et al. [5], the speed of rotation is data-driven for the LC-G model and does not suffer the potential ad hoc issue when determined by expert judgment [6]. It is worth mentioning that the impact of the so-called “accident hump” is more obvious for the Australian population, since ${\hat{b}}_{25,T+h}$ is lower than ${\hat{b}}_{x,T+h}$ of the neighboring ages. Such a difference, however, is much smaller when it is closer to the end of the forecasting period (2100).

**Figure 7:**
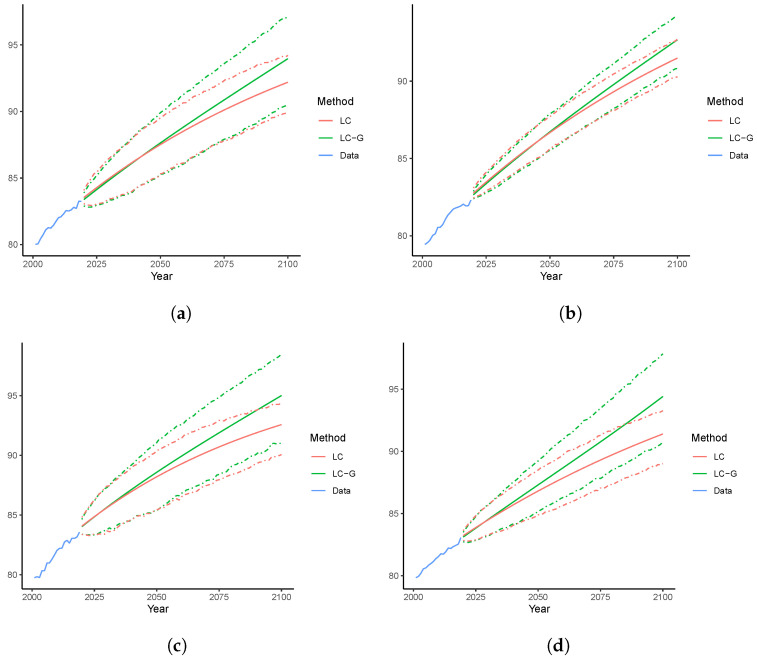
Forecast $e_{0}$ over 2020–2100, single-population models: (**a**) Australia; (**b**) Canada; (**c**) Spain; (**d**) Sweden.

Using those obtained ${\hat{b}}_{x,T+h}$, we can derive forecast mortality rates and thus produce the life expectancy using the LC-G model. Since life expectancy at birth ($e_{0}$) considers mortality rates at all ages, we contrast the forecast ${\hat{e}}_{0}$ of LC-G and LC over 2020–2100. Both the point and interval forecasts of Australia, Canada, Spain, and Sweden are plotted in Figure 7. It can be seen that ${\hat{e}}_{0}$ generated by LC-G are uniformly larger than those of the LC model. This is consistent with the fact that the LC-G model implements a rotation [5] of ${\hat{b}}_{x}$, and with the argued age-coherent property of the forecast mortality rates in the long run [6]. In addition, despite the PIs of LC-G being slightly wider than those of LC, their widths are at similar levels. This may be explained by the fact that ${\hat{k}}_{T+h}$ are identical for both models. As of 2100, the point forecast ${\hat{e}}_{0}$ of LC-G has exceeded the upper bound of the 95% PI of LC for all countries except Australia, implying a significant difference. More specifically, the point forecast ${\hat{e}}_{0}$ of LC-G (LC) for Australia, Canada, Spain, and Sweden grow from 83.4, 82.9, 82.9, and 82.7, respectively, in 2020 to 94.4, 94.3, 94.9, and 94.6 (92.3, 91.8, 91.9, and 90.1), respectively, in 2100.

**Figure 8:**
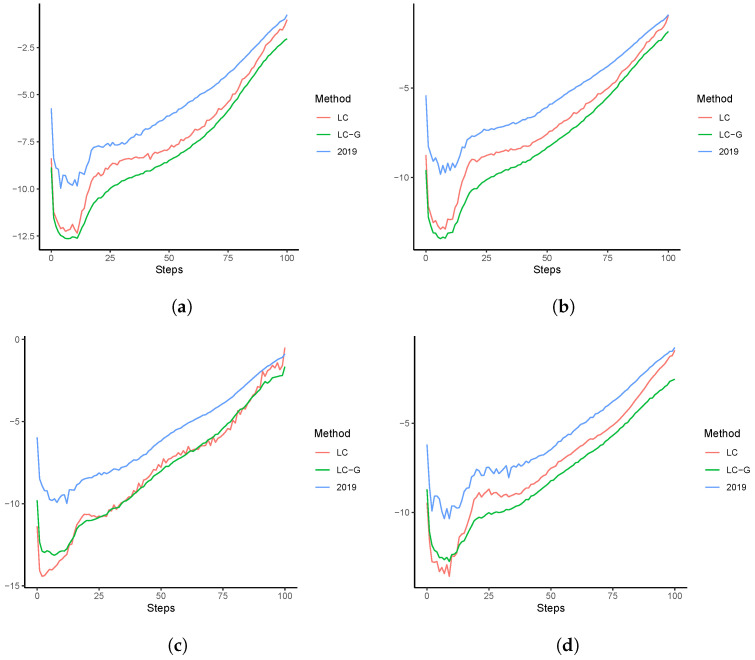
Forecast $\ln m_{x,t}$ over 2020–2100: (**a**) Australia; (**b**) Canada; (**c**) Spain; (**d**) Sweden.

To analyze the reasons contributing to those deviations in ${\hat{e}}_{0}$ of LC and LC-G in 2100, ranging from 2.1 (Australia) to 4.5 (Sweden) years, we plot the forecast logged mortality rates in Figure 8. Comparing with the true rates in 2019, it is observed that little improvements are gained at old ages for the forecasts produced by the LC model. In contrast, due to the rotation and age-coherent property, forecasts produced by LC-G demonstrate much more obvious mortality improvements at old ages. In addition, since ${\hat{b}}_{x,T+h}$ sum up to one at each step *h*, less mortality declines are distributed to the young ages. This contributes to the rotation-like difference when contrasting the $\ln{\hat{m}}_{x,T+h}$ produced by LC-G to those by LC. Overall, the difference in ${\hat{e}}_{0}$ produced by LC and LC-G, as presented in Figure 7, may be explained by the potentially large impacts of mortality rates at old ages in the calculation of life expectancy. Nevertheless, due to the influence of the smoothing penalties in (7), the long-run forecast logged mortality rates of LC-G are much smoother than those of the LC model.

## 4. Extensions to the Multi-Population Modelling: An Illustrative Example

A multi-population extension of the LC model is studied in the seminal work of Li and Lee [9]. An additional common factor which controls the relationships between populations is included in the proposed Li–Lee (LL) model. Specifically, the logged mortality rate is modeled as:

(8) $$\ln m_{x,t,j}=a_{x,j}+B_{x}K_{t}+b_{x,j}k_{t,j}+\varepsilon_{x,t,j},$$

where $a_{x,j}$ represents the average of the age-specific mortality level for the *j*th population, $B_{x}$ and $K_{t}$ represent the age effect and period effect of the common factor, $k_{t,j}$ is the time component of the *i*th population with age response $b_{x,j}$, and $\varepsilon_{x,t,j}$ is the population-specific error term. The common factor $B_{x}K_{t}$ describes the mortality trend of all modeled populations. To obtain the estimates, in this paper, we follow Li and Lee [9] to apply a usual LC model to the logged mortality rates averaged over all the fifteen populations. Consistent with LC, ${\hat{a}}_{x,j}$ is the average of $\ln m_{x,t,j}$ over *t*. The population-specific factor $b_{x,i}k_{t,i}$ can be estimated by applying SVD to the residual matrix $\left(\ln m_{x,t,j}-{\hat{a}}_{x,j}-{\hat{B}}_{x}{\hat{K}}_{t}\right)$.

Similar to the case under LC, the common mortality index $K_{t}$ is assumed non-stationary and can be modeled as a random walk with drift process. In contrast, the group-specific time component $k_{t,i}$ is fitted by a stationary autoregressive process (set to AR(1) in this paper, as considered in Li and Lee [9]), such that long-term forecasts are coherent (i.e., non-divergent) across the included populations. Given the data observed in the last year *T*, the *h*-step-ahead forecast of the logged mortality rate is given as follows:

(9) $$\ln{\hat{m}}_{x,T+h,j}={\hat{a}}_{x,j}+{\hat{B}}_{x}{\hat{K}}_{T+h}+{\hat{b}}_{x,j}{\hat{k}}_{T+h,j}.$$

Despite its desirable coherence among populations, $\ln{\hat{m}}_{x,T+h,j}$ of the LL model is still not age coherent. To see this, without identical ${\hat{B}}_{x}$ across ages, the non-stationary temporal term ${\hat{K}}_{T+h}$ in Equation (9) can lead to divergent long-run forecasts even for neighboring ages of the same population.

The multi-population extensions of the BMS, LC-E, and LC-G are straightforward to derive. Essentially, instead of fitting an LC model to forecast the common trend ${\hat{B}}_{x}{\hat{K}}_{T+h}$, BMS, LC-E, and LC-G models can be fitted for the averaged rates. Consequently, forecasts of their multi-population extensions can be produced below.

(10) $$\begin{array}{cc} \ln{\hat{m}}_{x,T+h,j}^{B}= & {\hat{a}}_{x,j}+{\hat{B}}_{x}{\hat{K}}_{T+h}^{B}+{\hat{b}}_{x,j}{\hat{k}}_{T+h,j}\\ \ln{\hat{m}}_{x,T+h,j}^{E}= & {\hat{a}}_{x,j}+{\hat{B}}_{x,T+h}^{E}{\hat{K}}_{T+h}+{\hat{b}}_{x,j}{\hat{k}}_{T+h,j}\\ \ln{\hat{m}}_{x,T+h,j}^{G}= & {\hat{a}}_{x,j}+{\hat{B}}_{x,T+h}^{G}{\hat{K}}_{T+h}+{\hat{b}}_{x,j}{\hat{k}}_{T+h,j} \end{array}$$

where the superscripts *B*, *E*, and *G* indicate the forecast of the multi-population extension of the BMS (LL-BMS), LC-E (LL-E), and LC-G (LL-G), respectively. It can be seen that in all cases, the population-specific average rate ${\hat{a}}_{x,j}$ and stationary trend ${\hat{b}}_{x,j}{\hat{k}}_{T+h,j}$ are unchanged from that of the LL model. For the LL-BMS, the same two adjustments, selecting an optimal sample period and using a Poisson model to obtain ${\hat{K}}_{T+h}^{B}$, as considered in the single-population BMS model are adopted for the averaged population. For the LL-E and LL-G models, ${\hat{B}}_{x,T+h}^{E}$ and ${\hat{B}}_{x,T+h}^{G}$ will be forecast using the constrained VAR model as for the single-population case described in Equation (6), based on the averaged population. The tuning parameters will be selected in the same way as described in Section 2.4.3, which will minimize the RMSFE of the averaged population for the hold-out sample.

Without imposing a long-run restriction, LL-BMS will lead to population-coherent forecasts as the LL, which are, however, not age coherent when h→∞. In contrast, following the discussions in Section 2.4, ${\hat{B}}_{x,T+h}^{E}$ and ${\hat{B}}_{x,T+h}^{G}$ are age invariant in the long run. Since ${\hat{k}}_{T+h,j}$ is a stationary process, ${\hat{b}}_{x,j}{\hat{k}}_{T+h,j}$ will converge to a constant for all ages. Thus, the logged mortality rates forecast by LL-E and LL-G are both age- and population-coherent in the long run across all ages and populations.

For illustration purposes, we consider the baseline results only as in Section 3.1 and Section 3.2 for the multi-population models. The RMSFE${}_{19}$ of the out-of-sample forecasts for each population are reported in Table 4. First, contrasting the results of Table 2 and Table 4, it is worth noting that in 10, 12, 8, and 9 out of the 15 countries, respectively, the forecasting performances of the LL, LL-BMS, LL-E, and LL-G are superior to those of the corresponding single-population counterparties. Second, LL-E and LL-G outperform the LL model in all the 15 populations. Compared to the LL-BMS model, LL-G and LL-H lead to more accurate forecasts in 11 cases. Finally, although LL-G beats LL-E in all cases except for Japan, the differences between the two time-varying models are still marginal in most cases. On average, LL-E and LL-G improve the forecasting performance of the LL by 9.1% and 11.3%, respectively. Thus, those results are very consistent with the single-population out-of-sample forecasting metrics, suggesting that allowing for dynamic age-specific mortality declines can produce the best-performing forecasts in the multi-population case.

**Figure 9:**
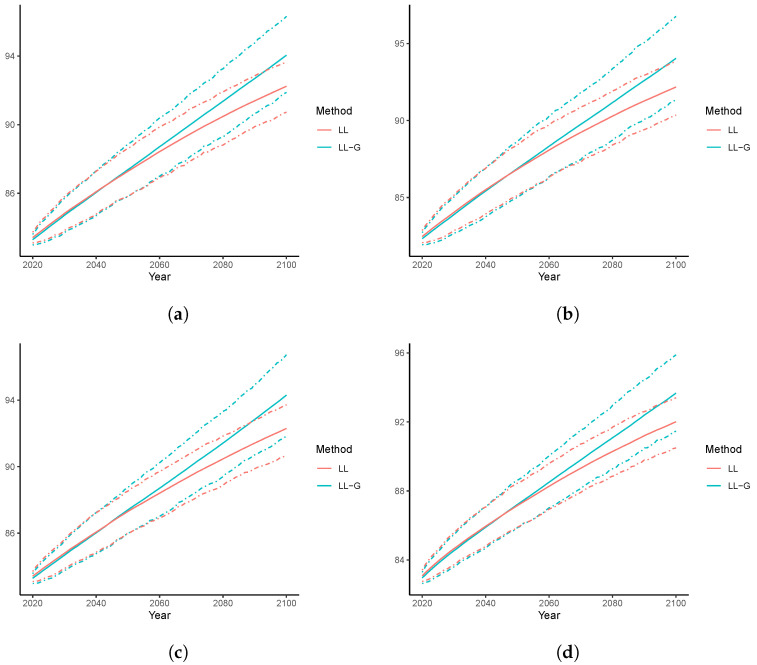
Forecast $e_{0}$ over 2020–2100, Multi-population models: (**a**) Australia; (**b**) Canada; (**c**) Spain; (**d**) Sweden.

We now examine the forecast life expectancy at birth over 2020–2100. In Figure 9, for the datasets of Australia, Canada, Spain, and Sweden, we present the point forecasts together with the 95% PIs produced by the LL and LL-G models. Consistent with Figure 7, the ${\hat{e}}_{0}$ generated by LL-G are uniformly larger than those by the LL. This can still be explained by the desirable age-coherence property of the LL-G model. In addition, all point forecasts of the LL-G model are above the corresponding upper bounds of the 95% PIs of the LC counterparty, suggesting a significant difference. Nevertheless, due to the population coherence, LL leads to similar forecasts of $e_{0}$ in 2100, ranging from 91.2 (Sweden) to 91.8 (Australia). For the same reason, the forecast $e_{0}$ in 2100 are also close to each other for the LL-G model, which vary from 93.0 (Sweden) to 94.8 (Canada). Nevertheless, for both the LL and LL-H models, the widths of the resulting PIs are narrower than those observed in Figure 7 for the single-population models. This can be attributed to the fact that more information is used in the multi-population framework, which can therefore reduce the uncertainty of the unexplained random disturbances.

## 5. Concluding Remarks

This paper investigates a time-varying coefficients extension of the Lee–Carter (LC) model. As argued in the influential work of Li et al. [5], mortality declines decelerate at young ages and accelerate at old ages. The lack of considering such dynamics makes the LC model less accurate in forecasting mortality rates in the long-term analysis.

To effectively resolve this issue without imposing computationally intensive burdens, we employ the kernel time-varying parametric approach as examined in a recent work of Chang and Shi [19]. Specifically, in-sample time-dependent $b_{x,t}$ are first estimated using the Epanechnikov (LC-E) and Gaussian (LC-G) kernel methods. To project the out-of-sample ${\hat{b}}_{x,T+h}$, a VAR-type model similar to that considered in Li and Lu [6] is further adopted. The forecasting of logged mortality rates is therefore conducted analogously to that for the LC model. For the proposed LC-E and LC-G models, we can draw five key conclusions in this paper. First, the long-run age-specific patterns of mortality declines are forced (asymptotically) identical across all ages. This ensures the desirable age coherence as proposed in the recent literature [6,19,28,29]. Second, the LC-E and LC-G models are computationally efficient and data-driven. Specifically, both models adopt the penalized least square, which results in closed-form solutions. In addition, the parameters and smoothing penalties relevant to the speeds of rotation in ${\hat{b}}_{x,T+h}$ are estimated/determined in a data-driven fashion, which is different from the ad hoc judgment of the rotation used in Li et al. [5]. Thirdly, using a large sample of uni-sex data of 15 countries over 1950–2019, we demonstrate the outstanding out-of-sample forecasting performance of the LC-E and LC-G models. The differences in the selection of the kernel function is deemed marginal. This conclusion is robust when a five-year instead of a one-year age mortality rate is modeled, and the training period is shortened to 1970–2000. Therefore, we recommend modelling and forecasting mortality rates with the time-varying coefficients LC model in demographic practices. Fourthly, our long-term analysis supports the effectiveness of the age-coherent forecasts produced by the recommended model. When forecast to 2100, significantly longer life expectancies at birth are generated by the LC-G model in most illustrated cases, which may be caused by its larger forecast mortality improvements at old ages. Finally, multi-population extensions of the LC-E (LL-E) and LC-G (LL-G) are straightforward to derive, which are based on the specification of the Li–Lee (LL) model Li and Lu [9]. On one hand, our proposed extension can achieve coherence across both ages and populations, whereas LL can only forecast non-divergent mortality rates across populations. On the other hand, consistent conclusions hold as those in the single-population scenario. Such conclusions include the more accurate out-of-sample forecasts and larger long-range projections of life expectancies led by the LL-G than the LL model.

There are some directions that are worth examining for future research. First, it is of interest to investigate female and male populations separately. For each country, differences between the selected tuning parameters of the LC-E and LC-G models may indicate important variations of the short-term speeds of mortality rotations for the two sexes. In addition, recent studies, including Vekas [46], have outlined that the rotation proposed by Li et al. [5] is notably more prevalent in populations of women than among men. This strongly motivates the exploration of sex differences using LC-E and LC-G models. Secondly, the fitted *d* in modelling the temporal patterns of $k_{t}$ may be made further time-dependent. Third, it is worth exploring the applicability of our specification to alternative models as listed in Section 2.5. For factor-based models, such as Renshaw and Haberman [34], our time-varying specification may be straightforwardly extensible. It will be more challenging to incorporate dynamics in other frameworks. Fourth, in the multi-population case, we may allow the population-specific $b_{x,i}$ to be dynamic in the forecasting steps. This more flexible specification might further improve the forecasting accuracy and provide additional information on the population-specific rotation of the $b_{x,i}$. Finally, impacts of COVID-19 may have significantly delayed mortality declines, especially for the old ages. After more data become available, our kernel-based models can be used to study how such shocks evolve and whether they are short-lived (and if so, when they eventually die out).

## Figures and Tables

**Table 1:** Unit root/Granger causality tests and bandwidths.

	LC-E			LC-G		
	ADF	GC	BW	ADF	GC	BW
Australia	3	4	5.07	3	3	3.73
Austria	6	5	3.13	5	5	2.18
Canada	3	6	5.23	3	6	3.55
Denmark	7	4	4.26	7	5	2.73
UK	3	3	9.79	3	3	9.81
Finland	3	3	5.15	3	3	1.55
France	4	3	3.37	4	4	2.27
Italy	4	4	3.21	4	5	2.18
Japan	6	7	9.71	6	6	9.88
Netherlands	5	6	5.96	5	7	4.18
Norway	5	4	5.23	5	4	3.73
Spain	5	3	3.21	5	3	2.18
Sweden	4	3	3.86	4	4	2.64
Switzerland	4	4	2.57	4	5	1.82
USA	4	4	9.92	4	4	8.18
Mean	4.4	4.2	-	4.3	4.5	-

Note: This table presents the augmented Dickey–Fuller (ADF) unit root and Granger causality test results, as well as the chosen bandwidths (BW). In all cases, we report the number of negative results (stationarity assumption does not hold for ADF, and VAR(1) is not adequate for GC) out of 101 age groups at 5% significant level.

**Table 2:** Connecting rod parameter of PUMA560 robot.

	RMSFE				Ranking			
Country	LC	BMS	LC-E	LC-G	LC	BMS	LC-E	LC-G
Australia	0.197	0.212	0.175	**0.175**	3	4	2	1
Austria	0.250	0.191	**0.183**	0.183	4	3	1	2
Canada	0.157	0.166	**0.140**	0.141	3	4	1	2
Denmark	0.389	0.413	**0.345**	0.345	3	4	1	2
UK	0.171	0.167	0.166	**0.164**	4	3	2	1
Finland	0.266	0.280	0.253	**0.248**	3	4	2	1
France	0.230	0.201	0.168	**0.168**	4	3	2	1
Italy	0.230	0.295	0.129	**0.129**	3	4	2	1
Japan	0.468	**0.354**	0.460	0.460	4	1	3	2
Netherlands	0.234	**0.220**	0.223	0.223	4	1	3	2
Norway	0.297	0.271	0.264	**0.264**	4	3	2	1
Spain	0.241	0.431	**0.163**	0.163	3	4	1	2
Sweden	0.244	0.249	**0.215**	0.215	3	4	1	2
Switzerland	0.359	0.331	**0.296**	0.296	4	3	1	2
USA	0.130	0.133	0.123	**0.122**	3	4	2	1
Mean	0.258	0.261	0.220	**0.220**	3	4	2	1

**Table 3:** Summary of robustness checks: Age and temporal variations.

	Mean	Std. Dev.	Median	Q${}_{1}$	Q${}_{3}$
Panel A: five-year age groups					
LC	0.256	0.089	0.239	0.198	0.288
BMS	0.265	0.090	0.237	0.202	0.326
LC-E	0.220	0.083	0.189	0.168	0.265
LC-G	**0.219**	**0.082**	**0.189**	**0.167**	**0.262**
Panel B: 1970–2000					
LC	0.240	0.080	0.218	0.192	0.262
BMS	0.239	0.084	0.220	0.180	0.288
LC-E	**0.200**	**0.062**	0.187	**0.153**	0.240
LC-G	0.200	0.062	**0.187**	0.152	**0.238**

Note: This table presents summary statistics of the root of mean squared forecasting error (RMSFE) with two variations in the sample: five-year age groups and period truncated to 1970–2019. Bold numbers are the smallest quantity for each statistic across the four models. Std. Dev. is the standard deviation. Q_1_ and Q_3_ are the first and third quartile, respectively.

**Table 4:** Multi-population out-of-sample forecasting performance.

	Error Measure				Ranking			
Country	LL	LL-BMS	LL-E	LL-G	LL	LL-BMS	LL-E	LL-G
Australia	0.185	0.179	0.176	**0.175**	4	3	2	1
Austria	0.244	0.234	0.214	**0.210**	4	3	2	1
Canada	0.163	**0.127**	0.143	0.136	4	1	3	2
Denmark	0.276	0.299	0.252	**0.250**	3	4	2	1
UK	0.167	**0.120**	0.158	0.153	4	1	3	2
Finland	0.268	**0.252**	0.263	0.260	4	1	3	2
France	0.195	0.204	0.147	**0.135**	3	4	2	1
Italy	0.233	0.226	0.217	**0.216**	4	3	2	1
Japan	0.184	**0.108**	0.168	0.169	4	1	2	3
Netherlands	0.179	0.165	0.151	**0.141**	4	3	2	1
Norway	0.247	0.244	0.233	**0.232**	4	3	2	1
Spain	0.271	0.268	0.249	**0.248**	4	3	2	1
Sweden	0.236	0.221	0.211	**0.198**	4	3	2	1
Switzerland	0.304	0.313	0.271	**0.263**	3	4	2	1
USA	0.185	0.175	0.171	**0.169**	4	3	2	1
Mean	0.222	0.209	0.202	**0.197**	4	3	2	1

## Data Availability

Not applicable.
